# The effect of folic acid, protein energy and multiple micronutrient supplements in pregnancy on stillbirths

**DOI:** 10.1186/1471-2458-11-S3-S4

**Published:** 2011-04-13

**Authors:** Aamer Imdad, Mohammad Yawar Yakoob, Zulfiqar A Bhutta

**Affiliations:** 1 Division of Women & Child Health, The Aga Khan University, Stadium Road, P.O. Box 3500, Karachi-74800, Pakistan

## Abstract

**Background:**

Pregnancy is a state of increased requirement of macro- and micronutrients, and malnourishment or inadequate dietary intake before and during pregnancy, can lead to adverse perinatal outcomes including stillbirths. Many nutritional interventions have been proposed during pregnancy according to the nutritional status of the mother and baseline risk factors for different gestational disorders. In this paper, we have reviewed three nutritional interventions including peri-conceptional folic acid supplementation, balanced protein energy supplementation and multiple micronutrients supplementation during pregnancy. This paper is a part of a series to estimate the effect of interventions on stillbirths for input to Live Saved Tool (LiST) model.

**Methods:**

We systematically reviewed all published literature to identify studies evaluating effectiveness of peri-conceptional folic acid supplementation in reducing neural tube defects (NTD), related stillbirths and balanced protein energy and multiple micronutrients supplementation during pregnancy in reducing all-cause stillbirths. The primary outcome was stillbirths. Meta-analyses were generated where data were available from more than one study. Recommendations were made for the Lives Saved Tool (LiST) model based on rules developed by the Child Health Epidemiology Reference Group (CHERG).

**Results:**

There were 18 studies that addressed peri-conceptional folic acid supplementation for prevention of neural tube defects (NTDs). Out of these, 7 studies addressed folic acid supplementation while 11 studies evaluated effect of folic acid fortification. Pooled results from 11 fortification studies showed that it reduces primary incidence of NTDs by 41 % [Relative risk (RR) 0.59; 95 % confidence interval (CI) 0.52-0.68]. This estimate has been recommended for inclusion in the LiST as proxy for reduction in stillbirths. Pooled results from three studies considered to be of low quality and suggest that balanced protein energy supplementation during pregnancy could lead to a reduction of 45% in stillbirths [RR 0.55, 95 % CI 0.31-0.97]. While promising, the intervention needs more effectiveness studies before inclusion in any programs. Pooled results from 13 studies evaluating role of multiple micronutrients supplementation during pregnancy showed no significant effect in reducing stillbirths [RR = 0.98; 95% CI: 0.88 – 1.10] or perinatal mortality [RR = 1.07; 95% CI: 0.92 – 1.25; random model]. No recommendations have been made for this intervention for inclusion in the LiST model.

**Conclusions:**

Peri-conceptional folic acid supplementation reduces stillbirths due to NTDs by approximately 41%, a point estimate recommended for inclusion in LiST.

## Background

The nutritional status of a woman before and during pregnancy is important for a healthy pregnancy outcome [1]. Pregnancy is a state of increased requirement of macro and micronutrients, and malnourishment or inadequate dietary intake before and during pregnancy, can lead to adverse perinatal outcomes [2,3].

Stillbirth is an important adverse outcome of pregnancy. Global estimates suggest that at least 3.2 million stillbirths occur annually [4], with 98% of these occurring in the developing world [5]. The risk factors for stillbirths in the low-/middle-income countries are myriad, and a systematic review by Di Mario et al. concluded that poor maternal nutritional status is one of the five factors significantly associated with stillbirths [6].

Many nutritional interventions have been proposed for pregnant mothers. These include multiple micronutrients (MMN), iron/folate, balanced protein energy, calcium, zinc and folic acid supplementation [7-11]. Some of these interventions are recommended universally for all women while some are proposed in the context of the nutritional status of mothers which may vary in different populations [3,12]. For example calcium is given during pregnancy for prevention of gestational hypertensive disorders but is effective only in populations with low baseline calcium intake [8,13]. Similarly iodine supplementation is effective in populations with iodine deficiency only.

In this review, our intention is to assess the evidence of the impact of three different nutritional interventions during pregnancy on stillbirths. We have reviewed the effect of peri-conceptional folic acid supplementation, balanced protein energy supplementation and multiple micronutrient supplements during pregnancy. This selection was based on an existing review of nutritional interventions for the prevention of stillbirths [14]. and only those interventions have been selected that have a proven benefit for reducing stillbirths or have a strong biological plausibility and now we review them in more depth. Calcium supplementation during pregnancy has been reviewed in another paper for this supplement [15]. This paper is a part of a series of papers to estimate effectiveness of an intervention for input into the Lives Saved Tool (LiST) [16]. The process of generating recommendations for an intervention involve qualitative evaluation of available evidence according to Grading of Recommendations, Assessment, Development and Evaluation (GRADE) criteria [17] and quantitative evaluation according to Child Health Epidemiology Reference Group (CHERG) rules [16]. For more details of the review methods, the adapted GRADE approach or the LiST model, see the CHERG method’s paper [16]. The following are the objectives of this review.

1. To estimate the effectiveness of peri-conceptional folic acid supplementation in reducing neural tube defects (NTDs) related stillbirths.

2. To estimate the effectiveness of balanced protein energy supplementation during pregnancy in reducing all-cause stillbirths.

3. To estimate the effectiveness of multiple micronutrient supplementation during pregnancy in reducing all-cause stillbirths.

## Methods

### Search

We systematically reviewed all published literature to identify studies addressing peri-conceptional folic acid supplementation, balanced protein energy and multiple micronutrient supplements during pregnancy. The search strategies used for the above mentioned nutritional interventions on PubMed are given as appendices 1, 2 and 3 respectively in Additional File 1. The last date of the search was 3^rd^ March 2010. Initial search identified titles and abstracts relevant to the interventions of interest. Full texts were then retrieved for selected studies for final inclusion and for data abstraction. We also reviewed the reference lists of identified articles, existing reviews and meta-analyses to identify studies that were not picked up in the main search. Authors were contacted for any additional data, if required.

### Inclusion/exclusion criteria

All randomized and quasi-randomized controlled trials assessing impact of peri-conceptional folic acid, balanced protein energy and multiple micronutrients supplements during pregnancy outcomes, were eligible for inclusion. Studies were included irrespective of language or publication status. We also reviewed observational studies for peri-conceptional folic acid supplementation as very few trials were conducted after huge protective effects of supplementation as shown in MRC trial [18]. Balanced protein energy supplementation was defined as nutritional supplementation during pregnancy in which proteins provided less than 25% of the total energy content [9]. Those studies were excluded in which the main intervention was simply dietary advice to pregnant women to increase protein intake, high protein supplementation (i.e. supplementation in which protein provided at least 25% or more of total energy content), isocaloric protein supplementation (where protein replaces an equal quantity of non-protein energy content), or low energy diets for pregnant women. Multiple micronutrients were defined as supplementation with at least five micronutrients and were compared with iron folic acid supplementation alone [7].

### Data abstraction and validity assessment

Every study that met the eligibility criteria was reviewed in detail and its characteristics abstracted into a standardized form [16]. The main variables extracted were country of the study, quality of allocation concealment, blinding status, characteristics of participants, sample size, description of intervention that included parameters like dose, frequency and duration of the supplements, and the follow-up period. All the studies were then graded according to the CHERG adaptation of the GRADE technique [16,17]. Each study was allocated a quality score of ‘high’ ‘moderate’ ‘low’ or ‘very low’. This assessment was based on the methodological quality of the study and consistency of results compared to that of other selected studies [16]. Any study getting a final score of ‘very low’ was excluded from the review [16,17]. The detailed data extraction with the limitation of studies is shown in Additional File 2.

### Quantitative data synthesis

We generated meta-analyses where data were available from more than one study. Dichotomous data were combined to get a pooled relative risk. In case where data from all the studies were not available in dichotomous form and risk ratios were available, meta-analysis was performed by generic inverse variance (GIV) method. This method is advantageous in these scenarios as it requires standard error (SE) and natural ‘log’ of effect size and data can be pooled without numerators and denominators [19]. For cluster randomized trials, we used the stated cluster adjusted relative risk and 95% confidence interval, irrespective of the method used. In case cluster adjustment was not done in the study, it was done by either adjusting the original sample size or inflating the SE times square of design effect [19]. Heterogeneity of the meta-analysis was assessed by visual inspection i.e. the overlap of the confidence intervals among the studies, and by the Chi square (P-value) of heterogeneity in the meta-analyses and I^2^ value. A low P value (less than 0.10) or a large chi-squared statistic relative to its degree of freedom (I^2^ >50 %) was considered as providing evidence of significant heterogeneity. In situations of substantial or high heterogeneity being present, causes were explored by sensitivity analysis. Fixed models were used for primary analysis and random models were used in case of significant heterogeneity in the pooled estimate. Results are presented as Mantel-Haenszel risk ratios (RR) and corresponding 95% confidence intervals (CI). All meta-analyses were conducted using software Review Manager Version 5 [20].

For recommendations to the LiST model, we summarized the evidence for each outcome including qualitative assessment of ‘overall’ evidence according to GRADE criteria and quantitative measures according to standard guidelines of the Child Health Epidemiological Review Group (CHERG) [16]. The qualitative evaluation of the overall (pooled) evidence was based on the volume and consistency of the evidence across studies, the size of pooled relative risk and the strength of the statistical evidence for an association between the intervention and the health outcome as reflected in the p-value [16].

## Results

### Peri-conceptional folic acid supplementation

**Figure 1:**
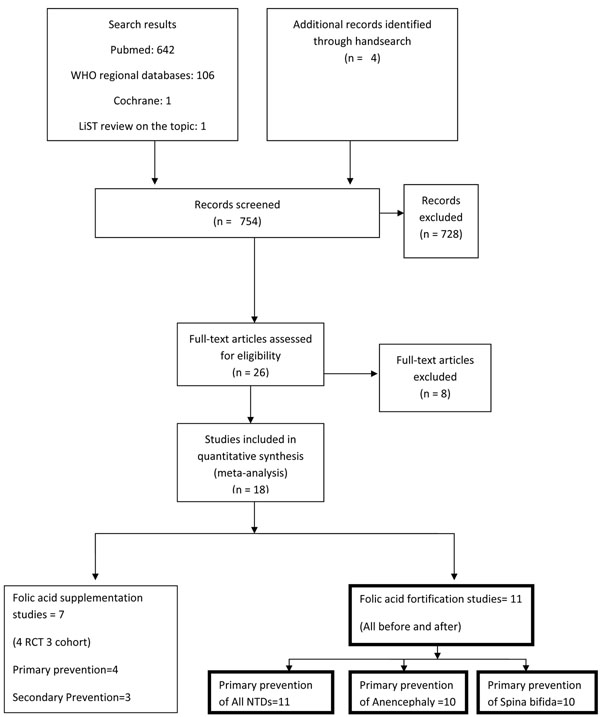
Flow diagram for identification of studies evaluating peri-conceptional Folic acid supplementation for prevention of stillbirths due to neural tube defects

The literature search identified 754 titles (Figure 1). After initial screenings of titles or abstracts and after duplicates were removed, we reviewed 26 studies. Eight studies were further excluded after review of full texts. Finally, data for eighteen studies were abstracted. One Cochrane [10] and one LiST review [21] were also available on the topic.

Out of eighteen selected studies, seven studies evaluated the effect of peri-conceptional folic acid supplementation [18,22-27] and eleven studies assessed fortification of food with folic acid [28-38]. All the fortification studies were before and after studies. Of the seven supplementation studies, there were four randomized controlled trials [18,23,24,26] and three cohort studies [22,25,27]. Four of the supplementation studies addressed primary prevention of neural tube defects [22,23,25,27] and three that of secondary preventions [18,24,26]. Meta-analysis of four studies (1 RCT and 3 cohort studies) for primary prevention of NTDs showed a reduction of 62 % in the incidence of NTDs [relative risk (RR) 0.38; 95 % confidence interval (CI) 0.29-0.51, I squared (I^2^) =27.9 %, fixed model] (data not shown). The overall quality grade for this consistent pooled estimate with 1 RCT [23] and 3 cohort studies [22,25,27] was that of ‘moderate’ level. Disaggregated data for stillbirth due to NTDs were not available from any of these studies. Pooled results of three RCTs for prevention of recurrent NTDs showed a reduction of 70 % in recurrence of NTDs [RR 0.30; 95 % CI 0.14-0.65, I^2^ =0 %, fixed model] (data not shown). As the evidence came from RCTs and the pooled estimate was consistent, the overall quality grade for this estimate was that of ‘high’ level. In these studies, disaggregated data for stillbirths due to NTDs were available from one study [24] with one stillbirth in the control group and no stillbirth in the intervention group, giving a relative risk of 0.17 (95 % CI 0.01-0.46).

**Figure 2:**
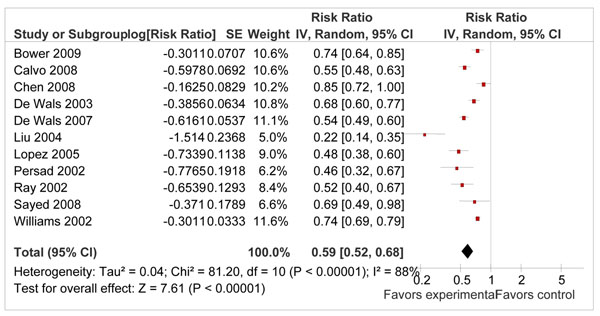
Effect of folic acid fortification on prevention of neural tube defects. Meta-analysis based on results of 11 before and after studies.

There were eleven before and after studies that addressed primary prevention of NTDs using folic acid fortification. Pooled results from these studies showed a reduction of 41 % in primary occurrence of NTDs [RR 0.59; 95 % CI 0.52-0.68, I^2^ =88 %, random model] (Figure 2). As there was a significant heterogeneity in the pooled estimate, random models were used. Ten of the included studies showed a clear benefit. The reason for significant heterogeneity was the difference in magnitude of ‘effect size’ of the included studies which ranged from a reduction of 26 % [33] to 78 % [38]. Although this evidence came from ‘low’ quality before and after studies, the overall quality grade for this estimate was that of ‘moderate’ level due to prominent protective effect in the same direction in most of the included studies. The disaggregated data for stillbirths due to NTDs was available from one study [31] with 5 stillbirths in intervention group and 29 in the control group giving a relative risk of 0.41 (95 % CI 0.16-1.07). This study from Canada found that before fortification (1992-1997), there were 29 stillbirths related to neural tube defects, out of a total of 531268 births, a cause-specific stillbirth rate of 5.5 per 100000 births. The corresponding figure after fortification (1998-2000) was 5 stillbirths out of 221 253, a rate of 2.3 per 100000.

Table 1 shows the summary of findings and qualitative assessment of evidence on folic acid supplementation/fortification in prevention of NTDs and related stillbirths. Keeping in mind the inadequacy of direct data on NTDs related stillbirth, we recommend an approximate reduction of 41% in NTD related stillbirths with an overall quality grade of “moderate”.

**Table 1:** Quality assessment of studies of peri-conceptional folic acid supplementation (by fortification) to prevent stillbirths from neural tube defects:

Quality Assessment				Directness		Summary of findings		
**No. of studies (Ref)**	**Design**	**Limitations**	**Consistency**	**Generalizability to Population of Interest**	**Generalizability to intervention of Interest**	**Events in intervention group**	**Events in control group**	**Relative Risk** **( 95 % CI)**

**NTDs related stillbirths: Low outcome specific quality**								

1	Before and after study	Only one study. Total event < 50 so cannot be considered for inclusion in the LiST	Only one study	Study from Canada	Folic acid fortification study	5	29	0.41 (0.16-1.07)a

**NTD incidence: Moderate outcome specific quality**								

**11**	Before and after	Low quality population based studies	Significant heterogeneity (I^2^ = 88%)	All studies from high-middle income countries	Folic acid fortification studies			0.59 (0.52-0.68)b

a: directly calculated from study data, b: pooled estimate calculated by generic inverse variance

### Balanced protein energy supplementation during pregnancy

**Figure 3:**
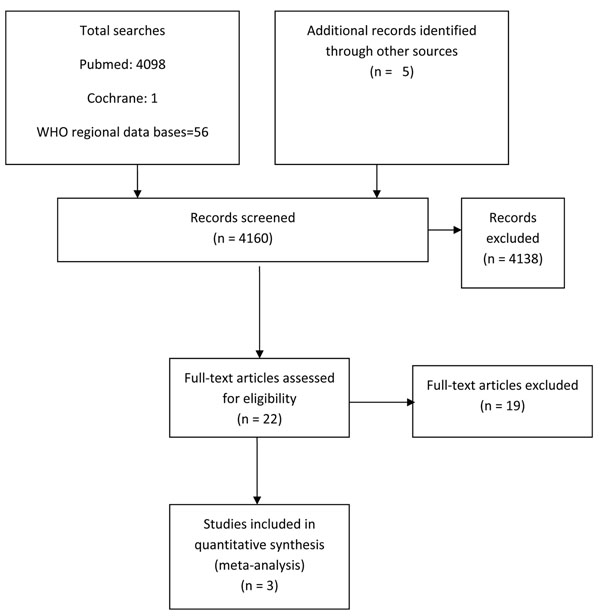
Flow diagram for identification of studies evaluating balanced protein energy supplementation during pregnancy for prevention of stillbirths

**Figure 4:**
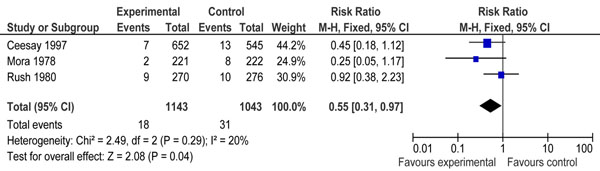
Effect of balanced protein energy supplementation during pregnancy on stillbirths

We identified 4160 titles from searches conducted in all databases (Figure 3). After screening the titles and abstracts, 22 studies were identified that addressed protein/energy supplementation during pregnancy. Eight of these studies were excluded because the intervention in these studies was either dietary advice about increase in protein/energy content, supplementation with isocaloric or high protein food [39-46]. Fourteen studies addressed balanced protein/energy supplementation during pregnancy [47-60]. Two of these studies were excluded because both the groups received food supplementation (high versus low energy) [53,54]. Another study was excluded because of ‘very low’ quality [60]. Eight studies were excluded because data for outcome of interest was not available [47-49,51,52,56,58,59]. Finally three studies were included in the review [50,55,57]. A Cochrane review was also available on the topic [9]. Two of the included studies were from developing countries [50,55] and one from a developed country [57]. The participants in all three studies were malnourished (as defined by authors). Pooled results from these three studies included a total of 2186 pregnancies and 49 stillbirths, showed that balanced protein energy supplementation during pregnancy leads to a significant reduction of 45% in all-cause stillbirths [RR 0.55, 95 % CI 0.31-0.97] (Figure 4). The overall grade quality of pooled data was that of ‘Low’ level (Table 2).

**Table 2:** Quality assessment of studies of balanced protein energy supplementation during pregnancy to prevent stillbirths

Quality Assessment				Directness		Summary of findings		
**No. of studies (Ref)**	**Design**	**Limitations**	**Consistency**	**Generalizability to Population of Interest**	**Generalizability to intervention of Interest**	**Events in intervention group**	**Events in control group**	**Relative Risk ( 95 % CI)**

**Stillbirth: Low outcome specific quality**								

**3**	RCT and quasi RCT	Methods of sequence generation and allocation concealment were not adequate in two of the included studies	Heterogeneity in the pooled data was not significant (I^2^=20%)	One of the study from developed country (USA); however, the participants in this study were from lower socioeconomic status	Different studies used different composition of formula for delivery of balance protein energy.	18	31	**0.55 (0.31-0.97)**

### Multiple micronutrient supplements during pregnancy

**Figure 5:**
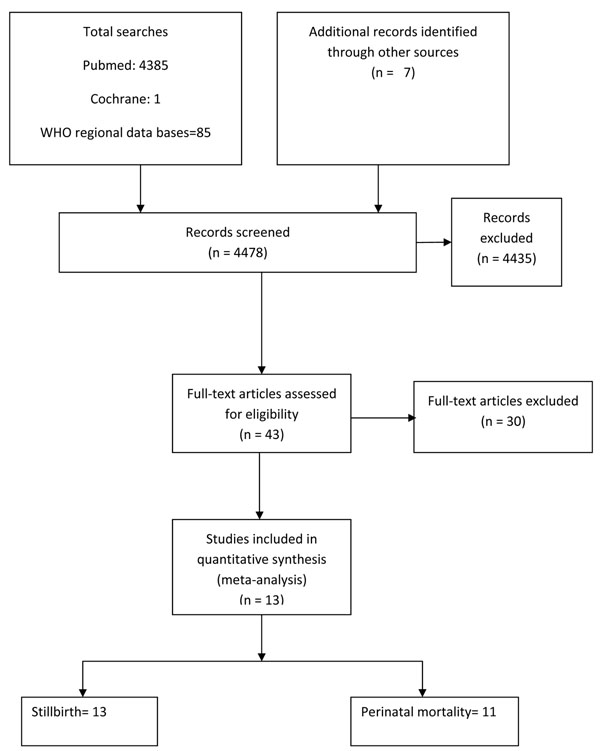
Flow diagram for identification of studies evaluating multiple micronutrient supplementations during pregnancy for prevention of stillbirths

**Figure 6:**
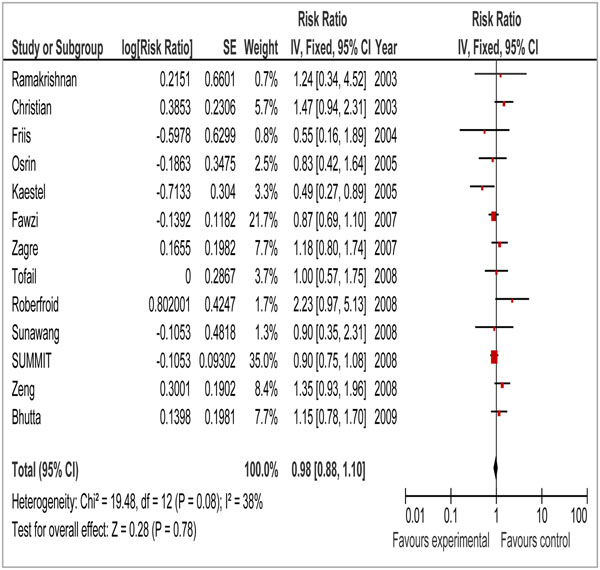
Impact of multiple micronutrients supplementation in pregnancy on stillbirths

A total of 4478 titles were identified from our search strategy (Figure 5). After screening the titles and abstracts, 13 studies were selected for inclusion in this paper [61-73]. Six of these 13 studies were cluster randomized trials [67-69,71,73,74]. Supplementation with multiple micronutrients failed to show a significant reduction in stillbirths when compared to iron folate supplementation (RR = 0.98; 95% CI: 0.88 – 1.10) (Figure 6). The impact on perinatal mortality was similar [RR 1.07, 95 % CI 0.92 –1.25] (Data not shown). As there was no significant effect of multiple micronutrient supplementation in reducing stillbirth or perinatal mortality, no recommendations have been made for LiST for this intervention (Table 3).

**Table 3:** Quality assessment of studies of multiple micronutrients supplementation during pregnancy to prevent stillbirths:

Quality Assessment				Directness		Summary of findings
**No. of studies (Ref)**	**Design**	**Limitations**	**Consistency**	**Generalizability to Population of Interest**	**Generalizability to intervention of Interest**	**Relative Risk ( 95 % CI)**

**Stillbirth: Low outcome specific quality**						

13	RCT	Methods of sequence generation and allocation concealment were not explicitly described in few of the studies	Heterogeneity in the pooled data was not significant (I^2^=38%)	All included studies from developing countries	Nine studies used UNIMMAP formulation (30 mg iron, 400 µg folic acid, 15 mg zinc, 2 mg copper, 65 µg selenium, 800 µg RE vitamin A, 1.4 mg vitamin B1, 1.4 mg vitamin B2, 18 mg niacin, 1.9 mg vitamin B6, 2.6 µg vitamin B12, 70 mg vitamin C, 5 µg vitamin D, 10 mg vitamin E and 150 µg iodine)	0.98 (0.88-1.10)a

**Peri-natal mortality: Low outcome specific quality**						

11	RCT	Methods of sequence generation and allocation concealment were not explicitly described in few of the studies	Significant heterogeneity (I^2^=56%). Random models used	All included studies from developing countries	Nine studies used UNIMMAP formulation (30 mg iron, 400 µg folic acid, 15 mg zinc, 2 mg copper, 65 µg selenium, 800 µg RE vitamin A, 1.4 mg vitamin B1, 1.4 mg vitamin B2, 18 mg niacin, 1.9 mg vitamin B6, 2.6 µg vitamin B12, 70 mg vitamin C, 5 µg vitamin D, 10 mg vitamin E and 150 µg iodine)	1.07 (0.92 –1.25)a

a: generic inverse variance

## Discussion

Evidence from a Cochrane review by Lumley et al. 2001 showed that peri-conceptional folic acid supplementation has a significant protective effect on occurrence of neural tube defects [RR 0.28, 95% CI 0.13–0.58], particularly in women who had a previous pregnancy affected by it (recurrent neural tube defects) [RR 0.31, 95% CI 0.14–0.66] [10]. A review by Blencowe et al. for Lives Saved Tool has shown that folic acid supplementation can reduce the primary incidence of NTDs by 62 % [RR 0.38; 95 % CI 0.29-0.51] and recurrence of NTDs by 70 %. [RR 0.30; 0.14-0.65] [21]. They also pooled data for fortification studies and showed a reduction of 46 % in primary incidence of NTDs. Our pooled estimates for primary prevention of neural tube defects by folic acid supplementation/fortification are similar to these reviews. The pooled estimate for folic acid fortification showed a reduction of 41 % (95 % CI 32 % to 48 %) in the occurrence NTDs. The small difference in effect size compared to previous LiST review was because we added three more studies to the previous met-analysis [28,30,32].

There was no convincing evidence from the current published literature in favor or against of peri-conceptional folic acid supplementation/fortification for prevention of stillbirths due to NTDs. Although it can be argued that a reduction in NTDs should be associated with a reduction in stillbirths, most of the studies did not report disaggregated data on proportion of stillbirths due to NTDs. Only one supplementation study [24] and one fortification study [31] reported direct data on NTD related stillbirths. We know from the previous literature that a major proportion of anencephalic babies and those with spina bifida cystica result in stillbirths [75-77]. Given the strong biological plausibility in favor of the intervention based on results of supplementation and fortification studies, we assumed that reduction in NTD incidence would be equal to reduction in NTDs related stillbirths with equal rates of incidence and fatalities of Anencephaly and Spina Bifida. It is important to mention that this estimate is applied to NTD related stillbirths only and the absolute effect of folic acid fortification for all-cause stillbirths will depend on the coverage of intervention and baseline incidence of NTDs in any given population.

We based our recommendations on folic acid fortification studies rather that of synthetic supplementation. This is based on the observation that widespread adoption of policies of folic acid supplementation in many developed countries have yielded disappointing results at a public health level [81]. The main contributing factors to this could be a relatively high proportion of unplanned pregnancies [82] and lack of easy access to a functioning health system and effective local social marketing interventions [23]. A policy of folic acid supplementation would be even more difficult to implement in low-income countries with high levels of poverty, poor health-care infrastructure and more number of unplanned pregnancies compared to developed countries [23,81,83,84]. Folic acid fortification seems a more suitable option for developing countries but it requires careful considerations including level of folic acid fortification and selection of suitable food vehicle. For example in certain population use of rice may be more common than flour or maize.

It is important to note that the effect of folic acid on incidence of NTDs and related stillbirths will be different in different countries. The amount of protective effect will depend on baseline NTDs incidence rate, folate deficiency in child bearing women, genetic susceptibility and existing system for screening and termination of affected pregnancies [75,78]. For example in one part of China, incidence of NTDs is much higher than other regions in the country and folic acid supplementation was more effective in reducing NTDs in this area compared to others [25]. The estimate in our meta-analysis of folic acid-fortification effect is based primarily on white populations and the effect may differ in different races. A before and after study from USA reported not only lower background NTD rates amongst black Americans compared to Hispanic or white groups, but also a reduced effect of folic acid fortification in the black American group [38]. This indicates that a policy of folic acid fortification may yield different results in different populations across the developing and developed countries.

The beneficial effects of folic acid may extend beyond NTDs and related stillbirths. A recent review by Blencowe et al. for Live Saved Tool has shown that folic acid fortification can reduce congenital anomalies related neonatal mortality by 13 % [21]. A study from Canada has shown that folic acid fortification reduced incidence of severe congenital heart diseases [79]. Another study has reported that folic acid supplementation can reduce spontaneous preterm delivery [80].

Balance protein energy supplementation has been shown to have a significant reduction on incidence of intrauterine growth restriction [2,9]. The current analysis suggests that it could also reduce occurrence of stillbirths [RR 0.55, 95 % CI 0.31-0.97]. There was no significant statistical heterogeneity in the pooled estimate (I^2^=20%) (Figure 5). The overall grade quality evidence for the pooled estimate was that of ‘low’ level due to inadequate method of sequence generation and allocation concealment of the two of the included studies. The total numbers of stillbirhs in all the three included studies was less than 50. According to CHERG rules, it is a pre-requisite for an estimate to be considered for inclusion in the LiST model that the total number of events is at least greater than 50 [16]. It should also be noted that all three included studies used different formulas to deliver the intervention. There is no single proven formula to recommend on large scale. Thus the value of protein supplementation is uncertain.

There was no effect of multiple micronutrient supplements on incidence of stillbirths compared to iron-folate supplementation alone. The Cochrane review on the subject by us in 2006 [7], however, did not have any meta-analysis on stillbirths as outcome. Most of the studies in the current analysis were representative of low- or middle-income populations. As the results were not statistically significant, we do not recommend MMN supplementation during pregnancy for inclusion in the LiST for reduction of stillbirths. A recently published review on MMN showed similar results [84].

In conclusion, folic acid fortification reduces incidence of neural tube defects and may also have an effect on stillbirths. This estimate of 41% reduction has been recommended as a proxy for reduction in stillbirths due to NTDs, for inclusion in the LiST model. Based on 3 studies balanced protein energy supplementation during pregnancy may reduce all-cause stillbirths by 45%. While promising, there is need of more operations research before we can recommend this intervention on large scale for reducing stillbirths. There is no evidence of effect of multiple micronutrients supplementation on reducing stillbirths.

## Competing interests

The authors declare no conflict of interest

## Authors’ contributions

Professor Zulfiqar A Bhutta developed the review parameters and secured support. Drs Aamer Imdad and Yawar Yakoob undertook the literature search, data extraction and analysis under the supervision of Professor Bhutta. Dr. Zulfiqar A. Bhutta gave advice in all the aspects of the project and was the overall supervisor.

## Supplementary Material

Additional File 1 The search strategies used for the above mentioned nutritional interventions on PubMed. Click here for file

Additional File 2 Data extraction sheet for studies included in the review Click here for file

## References

[B1] Maternal anthropometry and pregnancy outcomes. A WHO Collaborative Study: Introduction Bull World Health Organ 1995 73 Suppl 1 6 PMC2486652 20604475

[B2] de Onis M Villar J Gulmezoglu M Nutritional interventions to prevent intrauterine growth retardation: evidence from randomized controlled trials Eur J Clin Nutr 1998 52 Suppl 1 S83 93 9511024

[B3] Abu-Saad K Fraser D Maternal Nutrition and Birth Outcomes Epidemiol Rev 2010 20237078 10.1093/epirev/mxq001

[B4] Lawn JE Yakoob MY Haws RA Soomro T Darmstadt GL Bhutta ZA 3.2 million stillbirths: epidemiology and overview of the evidence review BMC Pregnancy Childbirth 2009 9 Suppl 1 S2 10.1186/1471-2393-9-S1-S2 19426465 PMC2679408

[B5] Smith GC Predicting antepartum stillbirth Curr Opin Obstet Gynecol 2006 18 6 625 630 10.1097/GCO.0b013e32801062ff 17099333

[B6] Di Mario S Say L Lincetto O Risk factors for stillbirth in developing countries: a systematic review of the literature Sex Transm Dis 2007 34 7 Suppl S11 21 17592385 10.1097/01.olq.0000258130.07476.e3

[B7] Haider BA Bhutta ZA Multiple-micronutrient supplementation for women during pregnancy Cochrane Database Syst Rev 2006 4 CD004905 17054223 10.1002/14651858.CD004905.pub2

[B8] Hofmeyr GJ Atallah AN Duley L Calcium supplementation during pregnancy for preventing hypertensive disorders and related problems Cochrane Database Syst Rev 2006 3 CD001059 16855957 10.1002/14651858.CD001059.pub2

[B9] Kramer MS Kakuma R Energy and protein intake in pregnancy Cochrane Database Syst Rev 2003 4 CD000032 14583907 10.1002/14651858.CD000032

[B10] Lumley J Watson L Watson M Bower C Periconceptional supplementation with folate and/or multivitamins for preventing neural tube defects Cochrane Database Syst Rev 2001 3 CD001056 11686974 10.1002/14651858.CD001056

[B11] Pena-Rosas JP Viteri FE Effects and safety of preventive oral iron or iron+folic acid supplementation for women during pregnancy Cochrane Database Syst Rev 2009 4 CD004736 19821332 10.1002/14651858.CD004736.pub3

[B12] Villar J Merialdi M Gulmezoglu AM Abalos E Carroli G Kulier R de Onis M Nutritional interventions during pregnancy for the prevention or treatment of maternal morbidity and preterm delivery: an overview of randomized controlled trials J Nutr 2003 133 5 Suppl 2 1606S 1625S 12730475 10.1093/jn/133.5.1606S

[B13] Trumbo PR Ellwood KC Supplemental calcium and risk reduction of hypertension, pregnancy-induced hypertension, and preeclampsia: an evidence-based review by the US Food and Drug Administration Nutr Rev 2007 65 2 78 87 10.1111/j.1753-4887.2007.tb00284.x 17345960

[B14] Yakoob MY Menezes EV Soomro T Haws RA Darmstadt GL Bhutta ZA Reducing stillbirths: behavioural and nutritional interventions before and during pregnancy BMC Pregnancy Childbirth 2009 9 Suppl 1 S3 10.1186/1471-2393-9-S1-S3 19426466 PMC2679409

[B15] Jabeen M Yakoob MY Imdad A Bhutta ZA Impact of interventions to prevent and manage pre-eclampsia on stillbirths BMC Public Health 2011 11 Suppl 3 S6 10.1186/1471-2458-11-S3-S6 PMC3231912 21501457

[B16] Walker N Fischer-Walker C Bryce J Bahl R Cousens S Standards for CHERG reviews of intervention effects on child survival Int J Epidemiol 2010 39 Suppl 1 i21 31 10.1093/ije/dyq036 20348122 PMC2845875

[B17] Atkins D Best D Briss PA Eccles M Falck-Ytter Y Flottorp S Guyatt GH Harbour RT Haugh MC Henry D Grading quality of evidence and strength of recommendations BMJ 2004 328 7454 1490 10.1136/bmj.328.7454.1490 15205295 PMC428525

[B18] Prevention of neural tube defects: results of the Medical Research Council Vitamin Study. MRC Vitamin Study Research Group Lancet 1991 338 8760 131 137 10.1016/0140-6736(91)90133-A 1677062

[B19] Higgins J, Green S Cochrane Handbook for Systematic Reviews of Interventions http://www.cochrane-handbook.org Version 5.0.2 2008 [updated September 2009]

[B20] RevMan The Cochrane Colloboration. Review Manager (RevMan) 5 for Windows 2003 Oxford, England

[B21] Blencowe H Cousens S Modell B Lawn J Folic acid to reduce neonatal mortality from neural tube disorders Int J Epidemiol 2010 39 Suppl 1 i110 121 10.1093/ije/dyq028 20348114 PMC2845867

[B22] Czeizel AE Dobo M Vargha P Hungarian cohort-controlled trial of periconceptional multivitamin supplementation shows a reduction in certain congenital abnormalities Birth Defects Res A Clin Mol Teratol 2004 70 11 853 861 10.1002/bdra.20086 15523663

[B23] Czeizel AE Dudás I Métneki J Pregnancy outcomes in a randomised controlled trial of periconceptional multivitamin supplementation: Final report Arch Gynecol Obstet 1994 255 131 139 10.1007/BF02390940 7979565

[B24] Kirke PN Daly LE Elwood JH A randomised trial of low dose folic acid to prevent neural tube defects. The Irish Vitamin Study Group Arch Dis Child 1992 67 12 1442 1446 10.1136/adc.67.12.1442 1489222 PMC1793975

[B25] Berry RJ Li Z Erickson JD Li S Moore CA Wang H Mulinare J Zhao P Wong LY Gindler J Prevention of neural-tube defects with folic acid in China. China-U.S. Collaborative Project for Neural Tube Defect Prevention N Engl J Med 1999 341 20 1485 1490 10.1056/NEJM199911113412001 10559448

[B26] Laurence KM James N Miller MH Tennant GB Campbell H Double-blind randomised controlled trial of folate treatment before conception to prevent recurrence of neural-tube defects Br Med J (Clin Res Ed) 1981 282 6275 1509 1511 10.1136/bmj.282.6275.1509 6786536 PMC1505459

[B27] Milunsky A Jick H Jick SS Bruell CL MacLaughlin DS Rothman KJ Willett W Multivitamin/folic acid supplementation in early pregnancy reduces the prevalence of neural tube defects JAMA 1989 262 20 2847 2852 10.1001/jama.262.20.2847 2478730

[B28] Bower C D'Antoine H Stanley FJ Neural tube defects in Australia: trends in encephaloceles and other neural tube defects before and after promotion of folic acid supplementation and voluntary food fortification Birth Defects Res A Clin Mol Teratol 2009 85 4 269 273 10.1002/bdra.20536 19180646

[B29] Calvo EB Biglieri A Impact of folic acid fortification on women's nutritional status and on the prevalence of neural tube defects Arch Argent Pediatr 2008 106 6 492 498 19107300 10.1590/S0325-00752008000600004

[B30] Chen BH Carmichael SL Selvin S Abrams B Shaw GM NTD prevalences in central California before and after folic acid fortification Birth Defects Res A Clin Mol Teratol 2008 82 8 547 552 10.1002/bdra.20466 18496833

[B31] De Wals P Rusen ID Lee NS Morin P Niyonsenga T Trend in prevalence of neural tube defects in Quebec Birth Defects Res A Clin Mol Teratol 2003 67 11 919 923 10.1002/bdra.10124 14745929

[B32] De Wals P Tairou F Van Allen MI Uh SH Lowry RB Sibbald B Evans JA Van den Hof MC Zimmer P Crowley M Reduction in neural-tube defects after folic acid fortification in Canada N Engl J Med 2007 357 2 135 142 10.1056/NEJMoa067103 17625125

[B33] Liu S West R Randell E Longerich L O'Connor KS Scott H Crowley M Lam A Prabhakaran V McCourt C A comprehensive evaluation of food fortification with folic acid for the primary prevention of neural tube defects BMC Pregnancy Childbirth 2004 4 1 20 10.1186/1471-2393-4-20 15450123 PMC524178

[B34] Lopez-Camelo JS Orioli IM da Graca Dutra M Nazer-Herrera J Rivera N Ojeda ME Canessa A Wettig E Fontannaz AM Mellado C Reduction of birth prevalence rates of neural tube defects after folic acid fortification in Chile Am J Med Genet A 2005 135 2 120 125 15846825 10.1002/ajmg.a.30651

[B35] Persad VL Van den Hof MC Dube JM Zimmer P Incidence of open neural tube defects in Nova Scotia after folic acid fortification CMAJ 2002 167 3 241 245 12186168 PMC117468

[B36] Ray JG Meier C Vermeulen MJ Boss S Wyatt PR Cole DE Association of neural tube defects and folic acid food fortification in Canada Lancet 2002 360 9350 2047 2048 10.1016/S0140-6736(02)11994-5 12504403

[B37] Sayed AR Bourne D Pattinson R Nixon J Henderson B Decline in the prevalence of neural tube defects following folic acid fortification and its cost-benefit in South Africa Birth Defects Res A Clin Mol Teratol 2008 82 4 211 216 10.1002/bdra.20442 18338391

[B38] Williams LJ Mai CT Edmonds LD Shaw GM Kirby RS Hobbs CA Sever LE Miller LA Meaney FJ Levitt M Prevalence of spina bifida and anencephaly during the transition to mandatory folic acid fortification in the United States Teratology 2002 66 1 33 39 10.1002/tera.10060 12115778

[B39] Anderson AS Campbell DM Shepherd R The influence of dietary advice on nutrient intake during pregnancy Br J Nutr 1995 73 2 163 177 10.1079/BJN19950021 7718538

[B40] Briley C Flanagan NL Lewis N In-home prenatal nutrition intervention increased dietary iron intakes and reduced low birthweight in low-income African-American women J Am Diet Assoc 2002 102 7 984 987 10.1016/S0002-8223(02)90225-7 12146565

[B41] Hankin ME Symonds EM Body weight, diet and pre-eclamptic toxaemia of pregnancy Australian and New Zealand Journal of Obstetrics and Gynaecology 1962 4 156 160 10.1111/j.1479-828X.1962.tb00198.x

[B42] Hunt IF Jacob M Ostegard NJ Masri G Clark VA Coulson AH Effect of nutrition education on the nutritional status of low-income pregnant women of Mexican descent Am J Clin Nutr 1976 29 6 675 684 1274890 10.1093/ajcn/29.6.675

[B43] Kafatos AG Vlachonikolis IG Codrington CA Nutrition during pregnancy: the effects of an educational intervention program in Greece Am J Clin Nutr 1989 50 5 970 979 2816804 10.1093/ajcn/50.5.970

[B44] Sweeney C Smith H Foster JC Place JC Specht J Kochenour NK Prater BM Effects of a nutrition intervention program during pregnancy. Maternal data phases 1 and 2 J Nurse Midwifery 1985 30 3 149 158 10.1016/0091-2182(85)90280-0 3847473

[B45] Iyengar L Effects of dietary supplements late in pregnancy on the expectant mother and her newborn Indian Journal of Medical Research 1967 55 85 89 6036057

[B46] Mardones-Santander F Rosso P Stekel A Ahumada E Llaguno S Pizarro F Salinas J Vial I Walter T Effect of a milk-based food supplement on maternal nutritional status and fetal growth in underweight Chilean women Am J Clin Nutr 1988 47 3 413 419 3279745 10.1093/ajcn/47.3.413

[B47] Atton C Watney PJM Selective supplementation in pregnancy: effect on birth weight Journal of Human Nutrition and Dietetics 1990 3 381 392 10.1111/j.1365-277X.1990.tb00248.x

[B48] Blackwell R Chow B Chinn K Blackwell B Hsu S Prospective maternal nutrition study in Taiwan: rationale, study design, feasibility and preliminary findings Nutrition Reports International 1973 7 517 532

[B49] Campbell Brown M Campbell DM Gillmer MDG Protein energy supplements in primigravid women at risk of low birthweight Nutrition in pregnancy Proceedings of the 10th Study Group of the RCOG 1983 London

[B50] Ceesay SM Prentice AM Cole TJ Foord F Weaver LT Poskitt EM Whitehead RG Effects on birth weight and perinatal mortality of maternal dietary supplements in rural Gambia: 5 year randomised controlled trial BMJ 1997 315 7111 786 790 9345173 10.1136/bmj.315.7111.786 PMC2127544

[B51] Elwood PC Haley TJ Hughes SJ Sweetnam PM Gray OP Davies DP Child growth (0-5 years), and the effect of entitlement to a milk supplement Arch Dis Child 1981 56 11 831 835 10.1136/adc.56.11.831 7030223 PMC1627387

[B52] Girija A Geervani P Rao GN Influence of dietary supplementation during pregnancy on lactation performance Journal of Tropical Pediatrics 1984 30 79 83 6726837 10.1093/tropej/30.2.79

[B53] Kardjati S Kusin JA De With C Energy supplementation in the last trimester of pregnancy in East Java: I. Effect on birthweight Br J Obstet Gynaecol 1988 95 8 783 794 10.1111/j.1471-0528.1988.tb06553.x 3048373

[B54] Lechtig A Habicht JP Delgado H Klein RE Yarbrough C Martorell R Effect of food supplementation during pregnancy on birthweight Pediatrics 1975 56 4 508 520 1165955

[B55] Mora J Navarro L Clement J Wagner M De Paredes B Herrera MG The effect of nutritional supplementation on calorieand protein intake of pregnant women Nutrition Reports International 1978 17 217 228

[B56] Ross S Nel E Naeye R Differing effects of low and high bulk maternal dietary supplements during pregnancy Early Human Development 1985 10 295 302 10.1016/0378-3782(85)90061-1 2985353

[B57] Rush D Stein Z Susser M A randomized controlled trial of prenatal nutritional supplementation in New York City Pediatrics 1980 65 4 683 697 6988785

[B58] Viegas OA Scott PH Cole TJ Eaton P Needham PG Wharton BA Dietary protein energy supplementation of pregnant Asian mothers at Sorrento, Birmingham. II: Selective during third trimester only Br Med J (Clin Res Ed) 1982 285 6342 592 595 10.1136/bmj.285.6342.592 6819029 PMC1499470

[B59] Viegas OA Scott PH Cole TJ Mansfield HN Wharton P Wharton BA Dietary protein energy supplementation of pregnant Asian mothers at Sorrento, Birmingham. I: Unselective during second and third trimesters Br Med J (Clin Res Ed) 1982 285 6342 589 592 10.1136/bmj.285.6342.589 6819028 PMC1499417

[B60] Tontisirin K Booranasubkajom U Hongsumarn A hewtong D Formulation and evaluation of supplementary foods for Thai pregnant women Am J Clin Nutr 1986 43 931 939 3521254 10.1093/ajcn/43.6.931

[B61] Fawzi WW Msamanga GI Urassa W Hertzmark E Petraro P Willett WC Spiegelman D Vitamins and perinatal outcomes among HIV-negative women in Tanzania N Engl J Med 2007 356 14 1423 1431 10.1056/NEJMoa064868 17409323

[B62] Friis H Gomo E Nyazema N Ndhlovu P Krarup H Kaestel P Michaelsen KF Effect of multimicronutrient supplementation on gestational length and birth size: a randomized, placebo-controlled, double-blind effectiveness trial in Zimbabwe Am J Clin Nutr 2004 80 1 178 184 15213046 10.1093/ajcn/80.1.178

[B63] Osrin D Vaidya A Shrestha Y Baniya RB Manandhar DS Adhikari RK Filteau S Tomkins A Costello AM Effects of antenatal multiple micronutrient supplementation on birthweight and gestational duration in Nepal: double-blind, randomised controlled trial Lancet 2005 365 9463 955 962 10.1016/S0140-6736(05)71084-9 15766997

[B64] Ramakrishnan U Gonzalez-Cossio T Neufeld LM Rivera J Martorell R Multiple micronutrient supplementation during pregnancy does not lead to greater infant birth size than does iron-only supplementation: a randomized controlled trial in a semirural community in Mexico Am J Clin Nutr 2003 77 3 720 725 12600867 10.1093/ajcn/77.3.720

[B65] Roberfroid D Huybregts L Lanou H Henry MC Meda N Menten J Kolsteren P Effects of maternal multiple micronutrient supplementation on fetal growth: a double-blind randomized controlled trial in rural Burkina Faso Am J Clin Nutr 2008 88 5 1330 1340 18996870 10.3945/ajcn.2008.26296

[B66] Tofail F Persson LA El Arifeen S Hamadani JD Mehrin F Ridout D Ekstrom EC Huda SN Grantham-McGregor SM Effects of prenatal food and micronutrient supplementation on infant development: a randomized trial from the Maternal and Infant Nutrition Interventions, Matlab (MINIMat) study Am J Clin Nutr 2008 87 3 704 711 18326610 10.1093/ajcn/87.3.704

[B67] Zagre NM Desplats G Adou P Mamadoultaibou A Aguayo VM Prenatal multiple micronutrient supplementation has greater impact on birthweight than supplementation with iron and folic acid: a cluster-randomized, double-blind, controlled programmatic study in rural Niger Food Nutr Bull 2007 28 3 317 327 17974365 10.1177/156482650702800308

[B68] Zeng L Dibley MJ Cheng Y Dang S Chang S Kong L Yan H Impact of micronutrient supplementation during pregnancy on birth weight, duration of gestation, and perinatal mortality in rural western China: double blind cluster randomised controlled trial BMJ 2008 337 a2001 10.1136/bmj.a2001 18996930 PMC2577799

[B69] Sunawang Utomo B Hidayat A Kusharisupeni Subarkah Preventing Low Birth Weight through Maternal Multiple Micronutrient Supplementation: A cluster-randomized controlled trial in Indramayu, West Java Food Nutr Bull 2009 in press 20120790 10.1177/15648265090304S403

[B70] Kaestel P Michaelsen KF Aaby P Friis H Effects of prenatal multimicronutrient supplements on birth weight and perinatal mortality: a randomised, controlled trial in Guinea-Bissau Eur J Clin Nutr 2005 59 9 1081 1089 10.1038/sj.ejcn.1602215 16015266

[B71] Shankar AH Jahari AB Sebayang SK Aditiawarman Apriatni M Harefa B Muadz H Soesbandoro SD Tjiong R Fachry A Effect of maternal multiple micronutrient supplementation on fetal loss and infant death in Indonesia: a double-blind cluster-randomised trial Lancet 2008 371 9608 215 227 10.1016/S0140-6736(08)60133-6 18207017

[B72] Christian P West KP Khatry SK Leclerq SC Pradhan EK Katz J Shrestha SR Sommer A Effects of maternal micronutrient supplementation on fetal loss and infant mortality: a cluster-randomized trial in Nepal Am J Clin Nutr 2003 78 6 1194 1202 14668283 10.1093/ajcn/78.6.1194

[B73] Bhutta ZA Rizvi A Raza F Hotwani S Zaidi S Soofi S Bhutta S A comparative evaluation of multiple micronutrient and iron-folate supplementation during pregnancy in Pakistan: impact on pregnancy outcomes Food Nutr Bull 2009 in press 10.1177/15648265090304S404 20120791

[B74] Christian P Khatry SK Katz J Pradhan EK LeClerq SC Shrestha SR Adhikari RK Sommer A West KP Jr. Effects of alternative maternal micronutrient supplements on low birth weight in rural Nepal: double blind randomised community trial BMJ 2003 326 7389 571 10.1136/bmj.326.7389.571 12637400 PMC151518

[B75] Botto LD Moore CA Khoury MJ Erickson JD Neural-tube defects N Engl J Med 1999 341 20 1509 1519 10.1056/NEJM199911113412006 10559453

[B76] Laurence KM Tew BJ Natural history of spina bifida cystica and cranium bifidum cysticum. Major central nervous system malformations in South Wales. IV Arch Dis Child 1971 46 246 127 138 10.1136/adc.46.246.127 4930541 PMC1647472

[B77] Obeidi N Russell N Higgins JR O'Donoghue K The natural history of anencephaly Prenat Diagn 2010 30 4 357 360 20198650 10.1002/pd.2490

[B78] Eichholzer M Tonz O Zimmermann R Folic acid: a public-health challenge Lancet 2006 367 9519 1352 1361 10.1016/S0140-6736(06)68582-6 16631914

[B79] Ionescu-Ittu R Marelli AJ Mackie AS Pilote L Prevalence of severe congenital heart disease after folic acid fortification of grain products: time trend analysis in Quebec, Canada BMJ 2009 338 b1673 10.1136/bmj.b1673 19436079 PMC2682153

[B80] Bukowski R Malone FD Porter FT Nyberg DA Comstock CH Hankins GD Eddleman K Gross SJ Dugoff L Craigo SD Preconceptional folate supplementation and the risk of spontaneous preterm birth: a cohort study PLoS Med 2009 6 5 e1000061 10.1371/journal.pmed.1000061 19434228 PMC2671168

[B81] Forrest JD Epidemiology of unintended pregnancy and contraceptive use Am J Obstet Gynecol 1994 170 5 Pt 2 1485 1489 8178895 10.1016/s0002-9378(94)05008-8

[B82] Oleary M Donnell RM Johnson H Folic acid and prevention of neural tube defects in 2000 improved awareness--low peri-conceptional uptake Ir Med J 2001 94 6 180 181 11495236

[B83] Vilaiphan P Suphapeetiporn K Phupong V Shotelersuk V An exceptionally low percentage of Thai expectant mothers and medical personnel with folic acid knowledge and peri-conceptional consumption urges an urgent education program and/or food fortification Int J Food Sci Nutr 2007 58 4 297 303 10.1080/09637480701217131 17566891

[B84] Ronsmans C Fisher DJ Osmond C Margetts BM Fall CH Multiple micronutrient supplementation during pregnancy in low-income countries: a meta-analysis of effects on stillbirths and on early and late neonatal mortality Food Nutr Bull 2009 30 4 Suppl S547 555 20120796 10.1177/15648265090304S409 PMC3428882

