# Supplementary material for: The effect of folic acid, protein energy and multiple micronutrient supplements in pregnancy on stillbirths
Source: BMC Public Health. 2011 Apr 13;11(Suppl 3):S4. doi: 10.1186/1471-2458-11-S3-S4 (PMC3231910; doi:10.1186/1471-2458-11-S3-S4)
Supplement: Additional File 1 — The search strategies used for the above mentioned nutritional interventions on PubMed. [file 1471-2458-11-S3-S4-S1.docx]

**Additional File 1**

**Appendix 1 Search strategy for peri-conceptional/Prenatal folic acid supplementation**

("folic acid"[MeSH Terms] OR "folic acid"[All Fields] OR "folic"[All Fields] OR "acid"[All Fields]) AND ("neonate"[All Fields]) OR “stillbirth” [All Fields] OR “fetal death” [All Fields] OR “intrauterine death” [All Fields] OR "mortality" [All Fields] OR "neonatal mortality" [All Fields]) AND (“neural tube defect” [All Fields] OR “neural” [All Fields] OR “tube” [All Fields]) AND ("pregnancy"[All Fields] OR "pregnancy"[MeSH Terms] OR periconceptual[All Fields] OR "peri conceptual[All Fields])

**Appendix 2 Search Strategy for Balanced Protein Energy Supplementation during pregnancy**

(Pregnancy* OR maternal OR "Mothers"[Mesh] OR "Pregnancy"[Mesh] OR "Pregnant Women"[Mesh] OR Stillbirth OR Intrauterine death) AND (balanced OR protein OR energy) AND (supplement*)

**Appendix 3: Search Strategy for Multiple Micronutrient Supplementations during Pregnancy**

("Mothers"[Mesh] OR "Pregnancy"[Mesh] OR mother* OR maternal OR pregnancy OR Stillbirth OR Intrauterine death) AND ("Micronutrients"[Mesh] OR "multiple micronutrient*" OR multivitamin OR micronutrient*) AND (supplement*)
